# Emergence of Different Recombinant Porcine Reproductive and Respiratory Syndrome Viruses, China

**DOI:** 10.1038/s41598-018-22494-4

**Published:** 2018-03-07

**Authors:** Yanyan Liu, Jianda Li, Jie Yang, Hao Zeng, Lihui Guo, Sufang Ren, Wenbo Sun, Zhi Chen, Xiaoyan Cong, Jianli Shi, Lei Chen, Yijun Du, Jun Li, Jinbao Wang, Jiaqiang Wu, Jiang Yu

**Affiliations:** 1 Shandong Key Laboratory of Animal Disease Control and Breeding, Institute of Animal Science and Veterinary Medicine, Shandong Academy of Agricultural Sciences, Jinan, 250100 China; 2 School of Life Sciences, Shandong University, Jinan, 250100 China; 3 School of Life Sciences, Shandong Normal University, Jinan, Jinan, 250014 China; 4 Biotechnology Research Center, Shandong Academy of Agricultural Sciences, Jinan, 250100 China

## Abstract

Epidemiological investigations were conducted on recently emerging porcine reproductive and respiratory syndrome virus (PRRSV) strains in Shandong province in 2014–2015. The proportion of the NADC30 strain identified by ORF7 sequence alignment has been gradually increasing. Three emerging PRRSV strains were successfully isolated, and the complete genomic sequences were determined. Our results indicate the importance of recombinant strains in Shandong province, China. There was a varied degree of recombination of two or three strains (classical, HP-PRRSV and/or NADC30). Moreover, the recombination strains affected the pathogenicity of newly emerged strains.

## Introduction

Porcine reproductive and respiratory syndrome (PRRS), a disease that is typically manifested by reproductive failures (e.g., late-term abortion, stillbirth and mummification) in pregnant sows and respiratory distress (e.g., interstitial pneumonia) in growing pigs, remains a substantial economic issue in the global swine industry^1–3^. Porcine reproductive and respiratory syndrome virus (PRRSV) is an enveloped, positive-sense, single-stranded RNA virus that belongs to the Arteriviridae family^4^. Its genome varies from 14.9 kb to 15.5 kb in length and possesses nine open reading frames (ORFs) that code seven structural proteins and 14 non-structural proteins^5^. The first two ORFs (ORF1a and ORF1b) encode an ORF1ab replicase polyprotein, and the remaining seven ORFs (ORF2a, 2b, and 3–7) code for minor structural proteins (GP2, GP3, and GP4) and major structural proteins (GP5, M, and N)^3^.

The initial characterization of circulating European genotype (genotype I, prototype virus Lelystad) and North American genotype (genotype II, prototype virus VR2332) isolates was found to be surprisingly genetically divergent. Although the overall disease phenotype, gross clinical symptoms, genomic organization and temporal emergence were all similar, these strains shared almost 60% homology at the nucleotide level. Genotype I was reported to be more virulent than genotype II in previous studies^6–8^.

PRRSV was first reported in North America in 1987^9^ and in Western Europe in 1990^10^. In China, PRRSV, belonging to genotype II, was first isolated in 1995^11,12^. In 2006, HP-PRRSV (highly pathogenic) first emerged from a less-pathogenic variant circulating in southern China and quickly spread throughout the country, causing considerable damage to the swine industry^13^. A second major HP-PRRSV outbreak occurred in 2009–2010, resulting in increased mortality and affecting a wider geographical area including several Southeast Asian countries^14^.

The most recent evolving episode was the appearance of the virulent PRRSV NADC30 strains in the United States in 2008; additionally, several NADC30-like strains were isolated in China, which showed the highest nucleotide similarity to a group of PRRSVs represented by NADC30^15–17^. These NADC30-like PRRSV strains included Henan-XINX (accession no. KF611905), HNyc15 (accession no. KT945018), and JL580 (accession no. KR706343). Moreover, several reports showed that the NADC30-like strains were now beginning to recombine with the Chinese HP-PRRSV in the field^18–20^, increasing the difficulty of the prevention and control of PRRS.

In our study, the detection of different PRRSV strains in 13 different regions of Shandong province since June 2014 was analyzed, and the positive samples of classical PRRSV, HP-PRRSV and NADC30 were counted by sequence analysis of ORF7. To fully understand the evolutionary patterns and dynamics of PRRSV and to aid prevention and control policies against the disease, a genomic scale analysis was necessary. Among all the PRRSV-positive samples, three recombination strains (SDhz1512, SDlz1601 and SDYG1606) were isolated, and their complete genomic sequences were determined. Moreover, the protein structure predictions and pathogenicity experiments of the three recombinant strains were compared. The recombination of the three strains was quite complex and demonstrated different degrees of recombination formed by two or three strains (NADC30, HP-PRRSV and/or classical PRRSV). The temporal changes in the relative genetic diversity of PRRSV were illustrated by Bayesian Skyline plots (BSP). Hence, our study offers a unique opportunity to investigate the evolution and spread of new genotype II PRRSV strains in Shandong province, China. Moreover, the findings play an important role in preventing and controlling the occurrence and spread of PRRSV.

## Results

### Epidemiology of PRRSV and variability of ORF7

PRRSVs of recent outbreaks (since 2014) are characterized by severe sow abortion and respiratory symptoms of conservation piglets. PRRSV strains were present in 13 different regions of Shandong province. Within a total of 347 clinical samples of diseased pigs (182 lung and lymph node samples and 165 serum samples), 243 samples were PRRSV-positive in RT-PCR using primers that targeted the PRRSV ORF7 (N) gene. The percentage of PRRSV positive samples was 40%-50% from June 2014 to April 2017 (Supplementary Fig. 1). Comparative analyses of ORF7 sequences showed that ORF7 primers were able to distinguish classical PRRSV (represented by the virus VR2332), HP-PRRSV (represented by the virus JXA1) and NADC30. However, the proportion of NADC30 strains in the PRRSVs of recent outbreaks (since 2014) was 50.62%, which was higher than that of classical PRRSV (6.67%) or HP-PRRSV (42.80%) (Table 1).

**Table 1:** Descriptive statistics for predictors tested for PRRS positive status for 13 different regions of Shandong province.

**Origin**	**No. of Clinical samples**	**No. of positive samples**	**Percent positive**	**Classical**	**HP-PRRSV**	**NADC30**
Linyi	115	94	81.74%	7	43	44
Jining	29	25	86.21%	1	13	11
Weifang	32	19	59.38%	0	8	11
Laiwu	19	11	57.89%	2	3	6
Yantai	17	8	47.06%	0	4	4
Zibo	14	9	64.29%	1	3	5
Binzhou	25	19	76.00%	1	7	11
Jinan	18	10	55.56%	1	5	4
Heze	13	13	100.00%	0	5	8
Dongying	17	9	52.94%	1	2	6
Taian	15	6	40.00%	0	4	2
Qingdao	12	7	58.33%	1	3	3
Liaocheng	21	13	61.90%	1	4	8
Total	347	243	70.03%	16/6.67%	104/42.80%	123/50.62%

**Figure 1:**
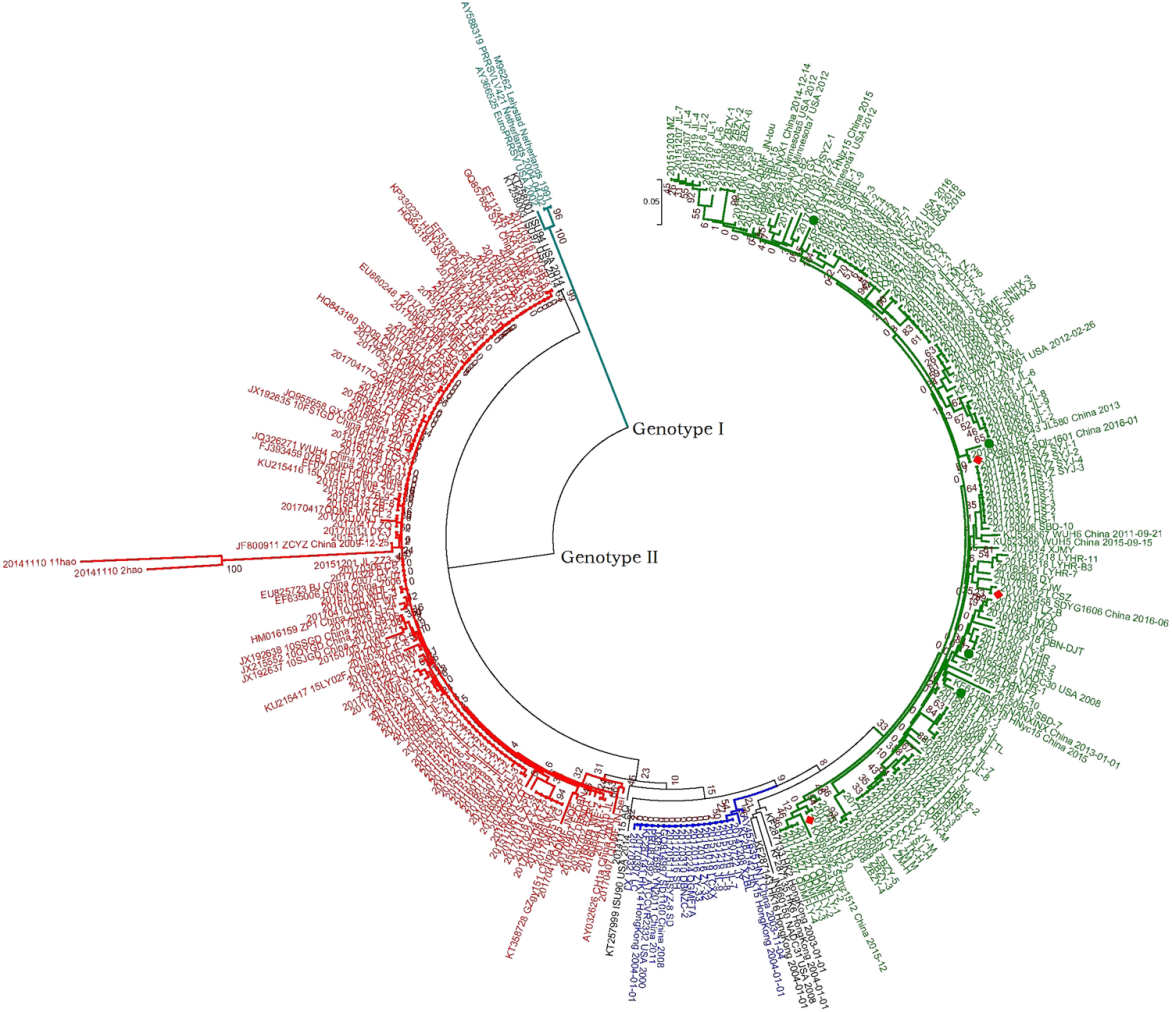
Phylogenetic tree based on the ORF7 gene of the 243 positive PRRSV and 54 reference viruses. Phylogenetic trees were constructed using the NJ method in MEGA7.0 with bootstrap values (1000 replicates), using the Kimura2-parameter model. The three isolates are marked with the red diamond.

To better understand the genetic relationship and evolution of PRRSV in Shandong province, China, an ORF7-based phylogenetic tree of 243 positive PRRSVs and 54 reference viruses was constructed. According to the phylogenetic tree (Fig. 1), these PRRSVs were divided into two distinct genotypes: genotype I and genotype II. All the PRRSVs of Shandong province belonged to genotype II, which was divided into three monophyletic lineages: the first sub-group contained 16 isolates belonging to classical PRRSV (represented by the virus VR2332), the second sub-group contained 104 isolates related to HP-PRRSV (represented by the virus JXA1), and the third sub-group contained 123 isolates (76 isolates from 2017) that were closely related to a group represented by NADC30.

### Identification of PRRSV isolation

**Figure 2:**
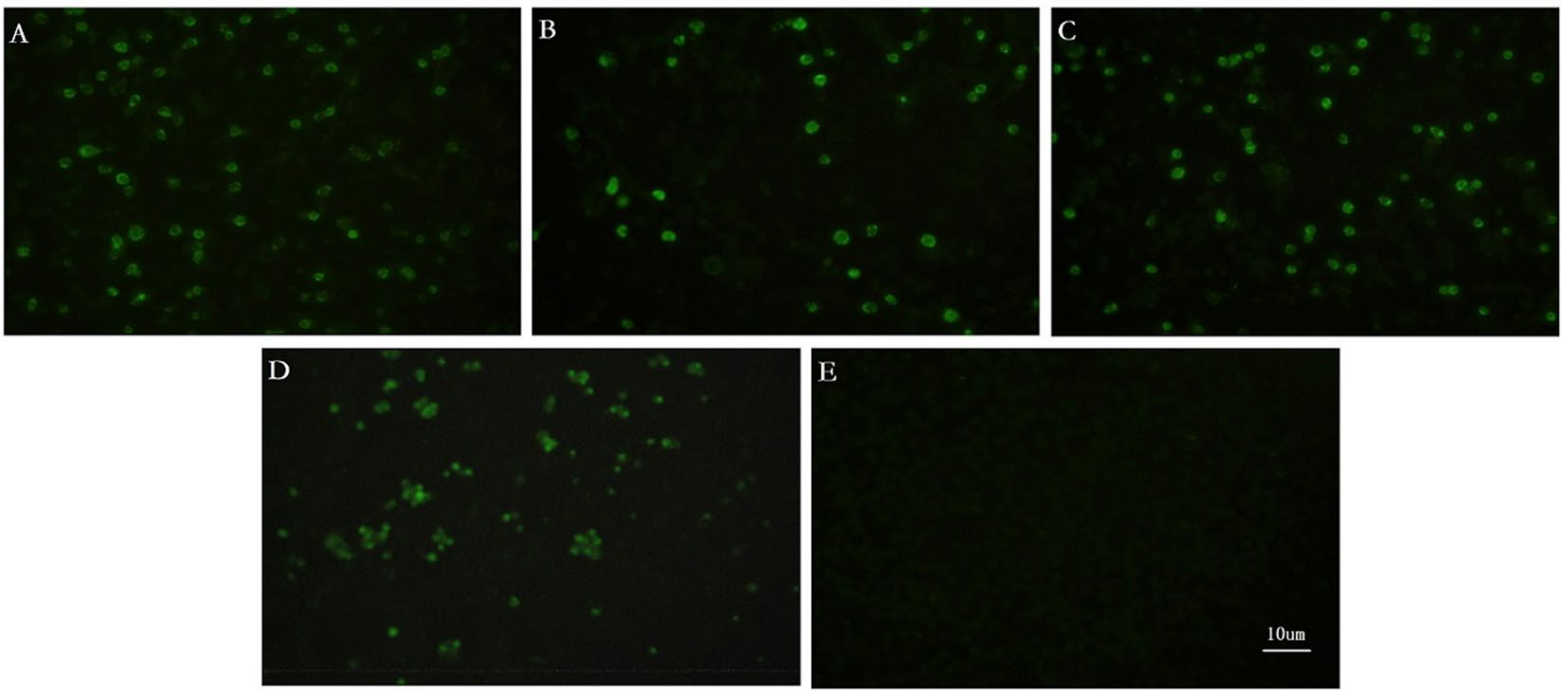
The results of the immunofluorescence assay. (**A**) PAMs inoculated with SDlz1601. (**B**) PAMs inoculated with SDYG1606. (**C**) PAMs inoculated with SDhz1512. (**D**) Positive control cells inoculated with SX-1. (**E**) PAM control cells.

Three PRRSV strains (SDhz1512, SDlz1601 and SDYG1606) that caused piglets to have clinical respiratory distress and interstitial pneumonia were recovered by PAMs. PAMs inoculated with four PRRSV strains (SDhz1512, SDlz1601, SDYG1606 and SX-1) showed green fluorescence, and the positive control cells infected with SX-1 had died. No fluorescence signal was observed in the control cells (Fig. 2).

### Genomic characterization of 3 PRRSV isolates

The complete genome sequences from three PRRSV isolates were determined and deposited in GenBank (accession no. KX980392, KX980393 and KY053458). They were classified as genotype II isolates but differed in length: 15069 nt for SDlz1601, 15367 nt for SDhz1512, and 15074 nt for SDYG1606. Full-length sequence analyses of SDlz1601, SDhz1512 and SDYG1606 were performed together with reference PRRSV strains including two North American isolates (VR2332 and NADC30) and one Chinese isolate (HP-PRRSV JXA1). The analysis showed that SDlz1601 shared 88.9% (JXA1), 84.5% (VR2332), 88.7% (NADC30) and 90.9% (JL580) nucleotide homology with the respective strains (data not shown); SDhz1512 shared 92.5% (JXA1), 89.8% (VR2332), 84.4% (NADC30), and 92.3% (HUN4) nucleotide homology with the respective strains (data not shown); and SDYG1606 shared 85.9% (JXA1), 89.8% (VR2332), and 91.5% (NADC30) nucleotide homology with the respective strains (Table 2). The nucleotide and amino acid homology of the 5′UTR, 3′UTR, ORF1a, ORF1b, and ORF2~7 genes in the three strains and the representative strains, including NADC30, VR2332 and JXA1, were compared. The results showed that the 5′UTR, ORF2~7 and 3′UTR of SDhz1512 shared 92.6~99.4% nucleotide (90.9~97.7% amino acid) homology with NADC30, which was higher than the homology shared with VR2332 and JXA1, whereas ORF1a of the SDhz1512 isolate shared 95.5% nucleotide (95.7% amino acid) identity with JXA1, which was higher than the others, and ORF1b of the SDhz1512 isolate shared 97.0% nucleotide (98.7% amino acid) identity with VR2332, which was also higher than the others. The SDlz1601 strain′s ORF1b, ORF2, ORF4, ORF6-7 and 3′UTR shared 91.1~97.5% nucleotide (90.2~97.7% amino acid) identity with NADC30, which was higher than the others, whereas 5′UTR, ORF1a, ORF3 and ORF5 shared 93.0~96.8% nucleotide (89.3~93.0% amino acid) identity with JXA1, which was higher than the other two strains. 3′UTR and ORF1b~ORF7 of SDYG1606 shared 87.0~97.3% nucleotide (90.9~98.0% amino acid) homology with NADC30; in addition, the 5′UTR region shared 97% nucleotide homology with the JXA1 strain (Table 2).

**Table 2:** Genome positions, protein sizes, and percentages of nucleotide and amino acid (in parenthesis) identity of SDlz 1601, SDhz 1512 and SDYG 1606 to other representative isolates (%).

**Region**	**Length**						**Identities (%) nucleotide (amino acid) sequence identity**								
	**JXA1**	**VR-2332**	**NADC30**	**SDlz1601**	**SDhz1512**	**SDYG1606**	**SDlz1601 to JXA1**	**SDhz1512 to JXA1**	**SDYG1606 to JXA1**	**SDlz1601 to VR2332**	**SDhz1512 to VR-2332**	**SDYG1606 to VR2332**	**SDlz1601 to NADC30**	**SDhz1512 to NADC30**	**SDYG1606 to NADC30**
Complete	15319	15451	15047	15069	15367	15074	88.9	92.5	85.9	84.5	89.8	83.6	88.7	84.4	91.5
5′UTR	188	190	191	189	189	189	96.8	92.6	97.3	89.5	93.1	90.5	89.5	99.4	91.0
ORF1a	7422(2475)	7512(2502)	7119(2375)	7119(2375)	7422(2475)	7119(2375)	87.3(87.6)	95.5(95.7)	82.0(82.9)	81.4(80.7)	87.0(85.8)	79.6(80.7)	83.3(85.1)	76.2(77.1)	87.9(89.3)
ORF1b	4383(1462)	4383(1462)	4383(1462)	4383(1462)	4383(1462)	4383(1462)	87.7(96.3)	92.5(96.5)	89.9(96.6)	87.4(95.5)	97.0(98.7)	88.6(96.2)	95.2(97.8)	89.0(96.4)	94.6(98.0)
ORF2	768(256)	768(256)	768(256)	768(256)	768(256)	768(256)	90.3(90.6)	86.5(87.5)	85.9(83.9)	89.5(88.6)	89.2(91.4)	89.4(86.7)	91.1(90.2)	95.4(94.9)	93.6(92.1)
ORF3	770(254)	770(254)	770(254)	770(254)	770(254)	770(254)	93.0(89.3)	82.0(78.7)	82.0(81.1)	88.0(87.4)	82.0(80.7)	84.0(83.4)	87.5(87.7)	95.0(94.0)	92.6(90.9)
ORF4	534(178)	534(178)	534(178)	534(178)	534(178)	534(178)	88.0(85.9)	87.0(84.8)	85.0(87.0)	86.8(85.9)	86.0(87.6)	87.2(87)	94.0(96.6)	96.6(97.1)	96.2(96.0)
ORF5	600(200)	600(200)	600(200)	600(200)	600(200)	600(200)	94.8(93.0)	83.8(84.5)	85.5(84.5)	87.3(85.5)	84.1(82.5)	85.5(83.0)	87.1(87.5)	92.5(92.5)	95.5(93.0)
ORF6	522(174)	522(174)	522(174)	522(174)	522(174)	522(174)	89.2(94.2)	89.2(94.2)	88.5(94.2)	90.6(94.2)	90.2(94.2)	89.4(94.2)	97.5(97.7)	97.5(97.7)	97.3(98.8)
ORF7	369(123)	369(123)	369(123)	369(123)	369(123)	369(123)	90.5(90.2)	90.5(91.8)	88.8(89.4)	91.0(91.8)	91.7(93.4)	91.0(92.6)	96.7(97.5)	97.2(97.5)	96.4(95.9)
3′UTR	150	151	151	149	151	152	86.7	87.4	88.8	90	90.7	90.7	97.3	97.3	97.3

### Amino acid analysis of NSP2, secondary structure and 3D-structure prediction

**Figure 3:**
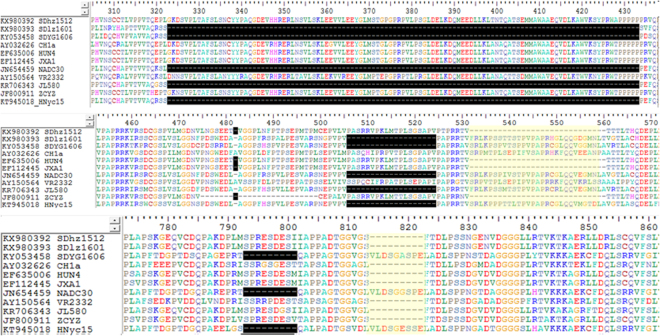
The sequences alignment of NSP2. Three discontinuous amino acid deletions (323–433, 482, and 506–524) in NSP2 of SDlz1601 and SDYG1606 and a 793–801 aa deletion in SDYG1606. Two NADC30-like PRRSVs (JL580 and HNyc15) and other representative PRRSVs including NADC30 (JN654459), JXA1 (EF112445), HUN4 (EF635006), VR2332 (U87392), and CH-1a (AY032626) were included in the analysis.

Non-structural protein 2 (NSP2) had the highest genetic diversity in the PRRSV genome. To further characterize the deletion regions, the NSP2 of the three isolated strains (SDhz1512, SDlz1601 and SDYG1606) was compared with 8 representative genotype II isolates. Multiple sequence alignment revealed that SDlz1601 and SDYG1606 had three discontinuous deletions (a total of 131 amino acids) in NSP2 that resembled previous NADC30-like PRRSVs, which can be used as molecular markers to distinguish them from other PRRSVs^15^. These deletions were not present in the Chinese HP-PRRSV strains (Fig. 3). Furthermore, the SDYG1606 isolate had the same 9 amino acid deletions (793–801) in NSP2 and was closely related to NADC30 (Fig. 3), but SDhz1512 had 30 amino acids deletion and was more similar to the HP-PRRSV strains (HUN4 and JXA1). Surprisingly, the 9 amino acid deletions (814~822) of NSP2 in SDlz1601 differed from those of previously sequenced NADC30-like isolates from China.

NSP2 secondary structures of the three isolates (SDhz1512, SDlz1601 and SDYG1606) and three reference strains (VR2332, JXA1 and NADC30) were predicted using protein software of DNAStar version 7.1, Madison WI (Supplementary Fig. 2). SDhz1512 was consistent with the highly pathogenic strain JXA1, in which the α-helix was deleted and the β-strand was increased. Compared with the classical strain VR2332, SDlz1601 and SDYG1606 were more consistent with the new NADC30-like strain, and the deletion of a total of 111 amino acids (323~433) resulted in the lack of a large α-helix, β-strand and random coil. In addition, the Antigenic Index in this region (320–360) was reduced to a low level using Protein software (DNAStar, version 7.1, Madison WI). In conclusion, the absence of amino acid groups in the discontinuous large fragments in NSP2 of NADC30-like strains resulted in significant changes in the secondary structure of the protein.

**Figure 4:**
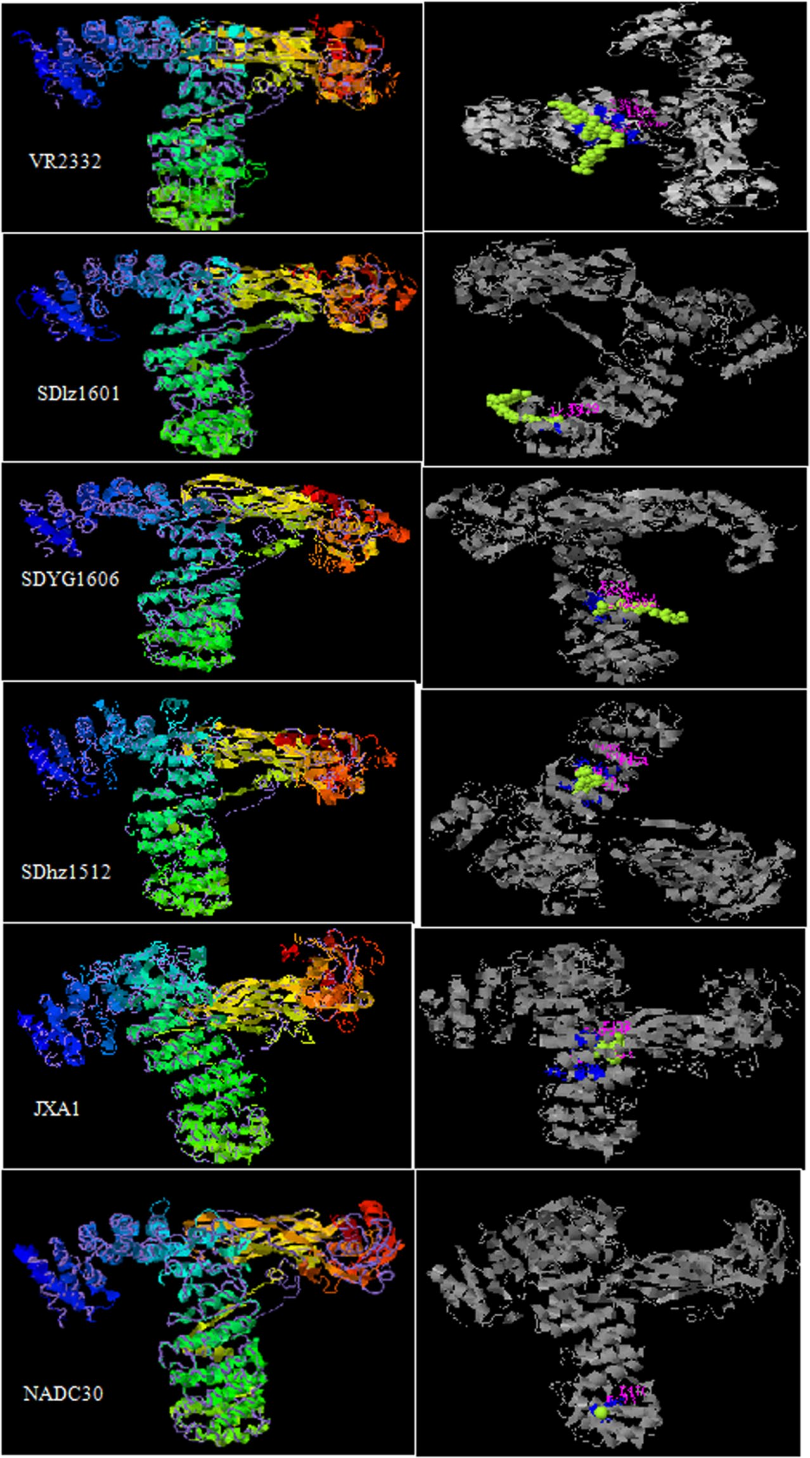
Tertiary structure models for NSP2 of VR2332, SDlz1601, SDYG1606, SDhz1512, JXA1 and NADC30 using I-TASSER Server. TM-score values range from [0, 1]. A higher score indicates a better structural match. Statistically, a TM-score <0.17 means a randomly selected protein pair with the gapless alignment taken from PDB; a TM-score >0.5 corresponds approximately to two structures of similar topology. C-score values are in the confidence interval [−5, 2], with higher values indicating better models. The TM-score and RMSD are the indexes for evaluating the molecular structure. The correlation of the C-score with RMSD and the TM-score is extra high.

The NSP2 amino acid sequences of the three isolated strains (SDhz1512, SDlz1601 and SDYG1606) and the three reference strains (VR2332, JXA1 and NADC30) were submitted to homology modeling by the I-TASSER Server^21,22^ for 3D-structure prediction. After the structure assembly simulation, I-TASSER uses the TM-align structural alignment program to match the first I-TASSER model to all structures in the PDB library. As a result, 5 models were established in all 5 strains, and the parameters of the first prediction model were the best. The parameters of VR2332 were as follows: TM-score 0.75, RMSD 10.8 and C-score −0.81. The parameters of the SDlz1601 strain were as follows: TM-score 0.65, RMSD 9.7 and C-score −0.50. The parameters of the SDYG1606 strain were as follows: TM-score 0.58, RMSD 11.0 and C-score −1.04. The parameters of the SDhz1512 strain were as follows: TM-score 0.65, RMSD 10.0 and C-score −0.51. The parameters of the JXA1 strain were as follows: TM-score 0.61, RMSD 10.0 and C-score −0.50. The parameters of the NADC30 strain were as follows: TM-score 0.65, RMSD 10.8, and C-score −0.81. The 3D spatial structure of the 6 virus strains was essentially the same, but the ligand binding sites differed (Fig. 4). The major amino acid residues are composed as follows: VR2332: R349, L352, T353, L356, S357, R364, E365, E366, D385, E386, D389, and Q390; JXA1: S371, L374, T404, M407, M408, V436, R439, K442, and S443; SDhz1512: S405, M408, A409, E413, P448, K451, P454, A455, G519, and V523; SDlz1601: T429, L433, A434, and D441; SDYG1606: P323, Q326, P327, A330, E331, S358, D362, and S366.

### Recombination analyses in SDhz1512, SDlz1601 and SDYG1606

In this study, a comprehensive and novel approach was employed to characterize recombination in the complete genome of PRRSV. Using seven algorithms, RDP, GENECONV, MaxChi, Chimaera, Bootscan, SiScanand, and 3Seq methods in RDP4.70 software provided strong evidence of recombination events in most of the sequences analyzed. Seventeen recombination events were detected in the three isolates (Table 3). SDYG1606 had 3 potential recombination signals, whereas SDhz1512 had 8 potential recombination signals and SDlz1601 had 6 potential recombination signals (Table 3). Of all 16 potential recombination events, 14 recombination events were detected in SDYG1606. SDlz1601 had a remarkably high degree of certainty, with a P-value in at least four algorithms of <1 × 10^−6^ (Table 3). The other 14 recombination events with a high degree of certainty due to a recombinant score >0.6 were located at 1–1818, 4823–8190, and 12098–12358 of SDYG1606 (events 1–3); 56–365, 1944–2021, 5732–6302, 7303–8111, 9088–11240, 12098–12358, and 11241–14635 of SDhz1512 (events 4–10); and 3004–7473, 12986–13638, 12057–12662, and 1944–2021 of SDlz1601 (events 13–15 and 17) (Table 3). In addition, the remaining 3 recombination events had a fair likelihood since their recombinant score was between 0.429 and 0.549 (Table 3). Furthermore, there were similar recombinant events with the same locations and parents in different isolates including a recombination located at 1944–2021 with parents NADC30/JXA1 in SDhz1512 (event 7) and SDlz1601 (event 17) (Table 3).

**Table 3:** Summary of possible recombination events in SDlz1601, SDhz1512 and SDYG1606 isolates identified by RDP4.

**Event number**	**Recombinant Sequence(s)**	**Breakpoint position(s)**	**Parental sequence**	**Recombinant score**	**P-Value for the six detection methods in RDP4**						
	**In Recombinant Sequence**	**Begin/End**	**Minor/Major**		**RDP**	**GENECONV**	**Bootscan**	**Maxchi**	**Chimaera**	**SiSscan**	**3Seq**
1	SDYG1606	4823/8190	JXA1/NADC30	0.697	7.94E-113	8.32E-116	5.58E-120	1.54E-42	4.35E-09	1.46E-50	2.16E-11
2	SDYG1606	1/1818	SX1/JL580	0.685	1.88E-152	4.56E-131	1.21E-136	2.56E-35	4.50E-34	NS	2.16E-11
3	SDYG1606	12098/12358	HNyc15/NADC30	0.65	7.37E-06	1.63E-04	NS	2.72E-04	2.10E-04	NS	2.42E-05
4	SDhz1512	9088/11240	HN1/SX09	0.673	NS	1.67E-76	8.97E-80	8.10E-15	1.70E-14	4.21E-32	NS
5	SDhz1512	5732/6302	SD1100/SX1	0.661	NS	5.35E-32	2.59E-34	7.42E-09	1.82E-13	7.25E-13	2.16E-11
6	SDhz1512	11241/14635	NADC30/JXA1	0.741	5.53E-110	NS	5.07E-90	9.09E-32	2.47E-09	4.85E-33	2.88E-11
7	SDhz1512	7303/8111	SD1100/JXA1	0.67	NS	1.85E-31	7.03E-35	2.17E-11	6.16E-12	9.15E-13	2.16E-11
8	SDhz1512	4702/14982	WUH5/SX1	0.624	2.02E-14	8.23E-08	2.46E-18	NS	NS	NS	1.44E-11
9	SDhz1512	56/365	VR2332/JXA1	0.617	2.27E-17	2.61E-12	2.18E-11	7.33E-04	2.05E-04	NS	6.48E-11
	SDhz1512	1944/2021	HNjz15/HLJB1	0.628	7.966E-10	3.40E-04	5.26E-03	NS	NS	NS	0.000249
10	SDhz1512	2062/3163	SX1/Unknown	0.429	2.03E-12	1.48E-20	1.09E-08	8.42E-07	7.58E-07	3.29E-13	7.20E-12
11	SDlz1601	360/2020	SX1/NADC30	0.549	4.12E-108	3.29E-113	1.53E-98	7.93E-38	1.58E-38	1.40E-39	NS
12	SDlz1601	3004/7473	JXA1/NADC30	0.629	1.36E-75	3.48E-87	2.41E-83	1.48E-44	1.77E-10	1.90E-64	3.78E-08
13	SDlz1601	12986/13638	JXA1/NADC30	0.674	4.37E-48	1.61E-40	1.28E-38	8.34E-14	3.55E-15	5.33E-16	1.44E-11
14	SDlz1601	12057/12662	TJbd141/NADC30	0.663	NS	5.92E-37	2.25E-44	1.01E-14	1.72E-12	6.58E-18	1.44E-11
15	SDlz1601	1/354	NVDCSDXX2013/JL580	0.549	7.19E-18	9.17E-12	1.61E-17	1.93E-02	3.91E-03	5.06E-04	NS
16	SDlz1601	1944/ 2021	NADC30/JXA1	0.628	7.966E-10	0.0003397	0.0052645	NS	NS	NS	0.000249

**Figure 5:**
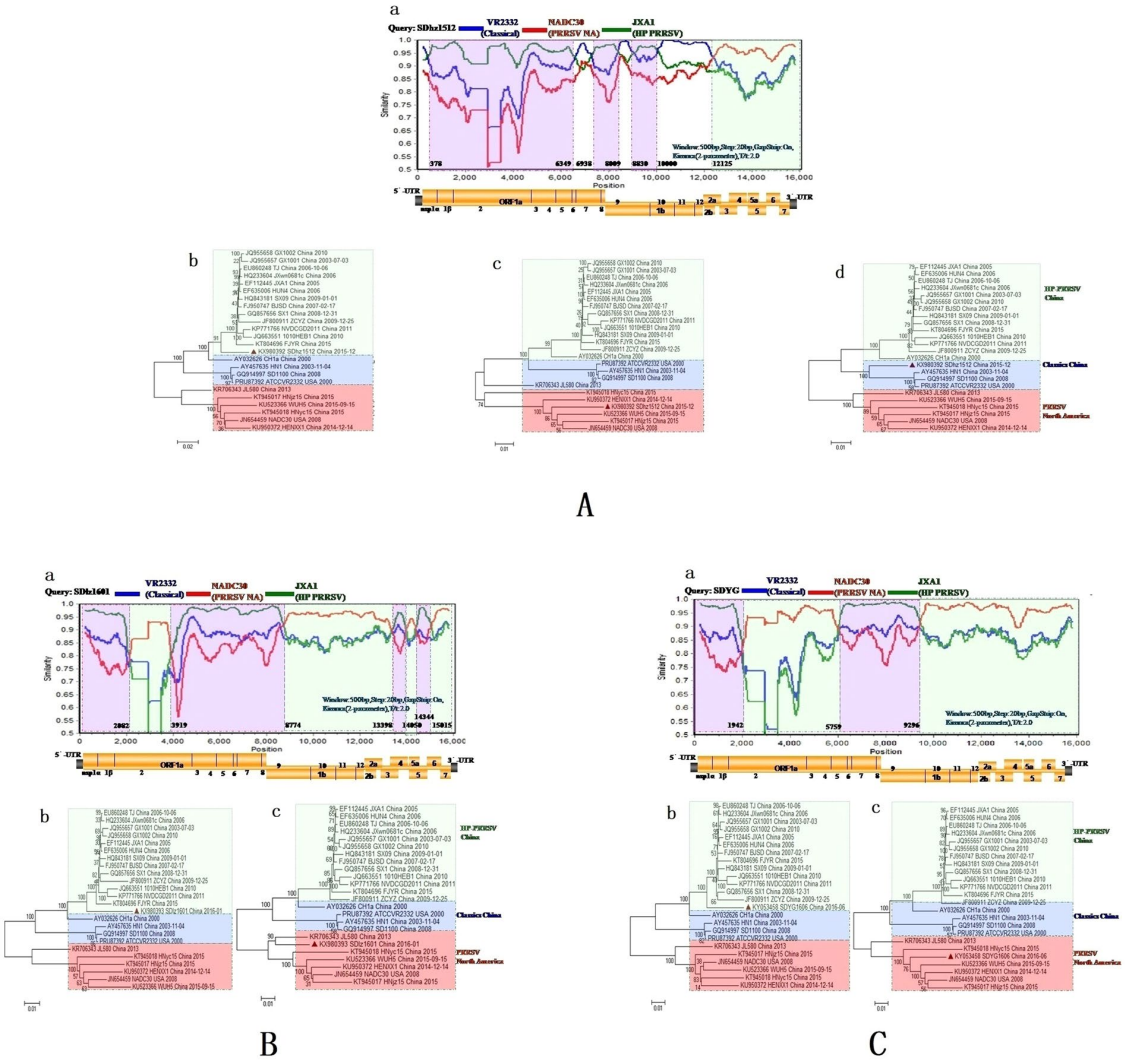
Recombination analysis of the three PRRSV strains. Recombination analysis of SDhz1512 (**A**) SDlz1601 (**B**) and SDYG1606 (**C**) using SimPlot with a sliding window of 500 nt, moving in 20 nt steps. Genome scale similarity comparisons of three PRRSV strains (query) with NADC30 (parental group A, red), JXA1 (parental group B green), and VR2332 (outgroup C, blue). Recombination breakpoints are shown as dotted lines, with the locations indicated at the bottom. The background color of parental region A (with reference to the NADC30 strain) is light green, whereas that of parental region B (HP-PRRSV JXA1) is shaded light purple and that of parental region C (VR2332) is shaded white. The phylogenies of parental region A(c), parental region B(b), and parental region C(d) are shown below the similarity plot.

To further validate and confirm the putative recombination events and their breakpoint positions in isolates detected with RDP, the SimPlot method in the SimPlot v3.5.1 program was employed. This method records the coherence of the sequence relationship over the entire length of the isolate and its potential parents^23^. This study revealed that SDhz1512, SDlz1601 and SDYG1606 were the result of recombination between the NADC30-like viruses and the classical strains and HP-PRRSV strains present in outbreaks in China. From the similarity plot, we identified seven recombination breakpoints in SDhz1512: one was located in NSP1α (nucleotides [nt] 352), two points were located in NSP7 (nt6349 and 6938), two points were located in NSP9 (nt8009 and 8830), one point was located in NSP10 (nt10000), and the last point was located in ORF2a (nt12125) (Fig. 5Aa). The analysis revealed that SDlz1601 was the result of recombination between the NADC30 viruses and HP-PRRSV strains circulating in China. There were seven recombination breakpoints detected in the SDlz1601 isolate: two points were located in NSP2 (nt 2082 and 3919), one was located in NSP9 (nt8774), two points were located in ORF4 (nt 13398 and 14050), one was located in ORF5 (nt14344), and one was located in ORF6 (nt15015) (Fig. 5Ba). The analysis revealed that SDYG1606 was the result of recombination between the NADC30 viruses and the classic HP-PRRSV strains circulating in China. From the similarity plot, we identified three recombination breakpoints: one was located in NSP2 (nt1942) and the others were located in NSP4 (nt5759) and NSP9 (nt9296) (Fig. 5Ca). The breakpoints of SDhz1512 separate the genome into eight regions: three are closely related to the HP-PRRSV strains in China (represented by JXA1) (Fig. 5Ab), four are closely related to classical PRRSV strains from China (represented by China1a) (Fig. 5Ac), and remaining region is closely related to the PRRSV strains from North America (represented by NADC30) (Fig. 5Ad). The breakpoints of SDlz1601 separate the genome into eight regions: four are closely related to the classic HP-PRRSV strains in China (represented by JXA1) (Fig. 5Bb) and the other four are closely related to PRRSV strains from North America (represented by NADC30) (Fig. 5Bc). The breakpoints of SDYG1606 separate the genome into four regions: two are closely related to the classic HP-PRRSV strains in China (represented by JXA1) (Fig. 5Cb) and the other two are closely related to the PRRSV strains from North America (represented by NADC30) (Fig. 5Cc).

Interestingly, eight of the above 6 reported and 3 isolated strains had different parents of recombination with other PRRSV strains such as classical strains (VR2332 and/or CH1a), HP-PRRSV (JXA1 and/or 09NEN1), and NADC30 (Table 4). The recombination breakpoints analysis of the above recombinants demonstrated that they were similar to the phenomenon of fracture recombination, and the recombinant positions mainly occurred in NSP2, NSP9, ORF2, ORF4 and ORF7 (Table 4). These proteins may have been the hot regions for recombination event breakpoints.

**Table 4:** Recombination analysis of nine PRRSV strains Simplot v3.5.1 software was used to analyze recombination.

Virus	Recombination with	Recombination sites	GenBank Access NO.	Isolation year	Isolation sites
SDYG1606	JXA1	NSP2, NSP5~NSP9	KY053458	2016	Shandong province
	NADC30	NSP2~NSP5, NSP9~ORF7			
SDhz1512	VR2332/CH1a	NSP1α, NSP6-7, NSP9, ORF1b, ORF2,	KX980392	2015	Shandong province
	NADC30	ORF2-7			
	JXA1	NSP1α-NSP6, NSP7-9, NSP9-ORF1b			
SDlz1601	JXA1	NSP2, NSP9, ORF3, ORF4, ORF5, ORF6	KX980393	2016	Shandong province
	NADC30	NSP2, NSP9~ORF3, ORF4~ORF5, ORF6~7			
JL580	NADC30	NSP2, NSP2~NSP3, NSP7~ORF2, ORF4~ORF7	KR706343.1	2014	Jilin province
	09HEN1	NSP2, NSP2~NSP7, ORF2~ORF4			
HNjz15	/	/	KT945017.1	2015	Henan province
HNyc15	VR2332/CH1a	ORF2-4	KT945018.1	2015	Henan province
FJXS15	JXA1	NSP2~ORF4	KX758250	2015	Fujian province
	NADC30	NSP2, ORF4~7			
HENAN-HEB	JXA1	NSP2	KJ143621.1	2013	Henan province
HENAN-XINX	VR2332	NSP2-5	KF611905.1	2013	Henan province

### Phylogenetic analysis

**Figure 6:**
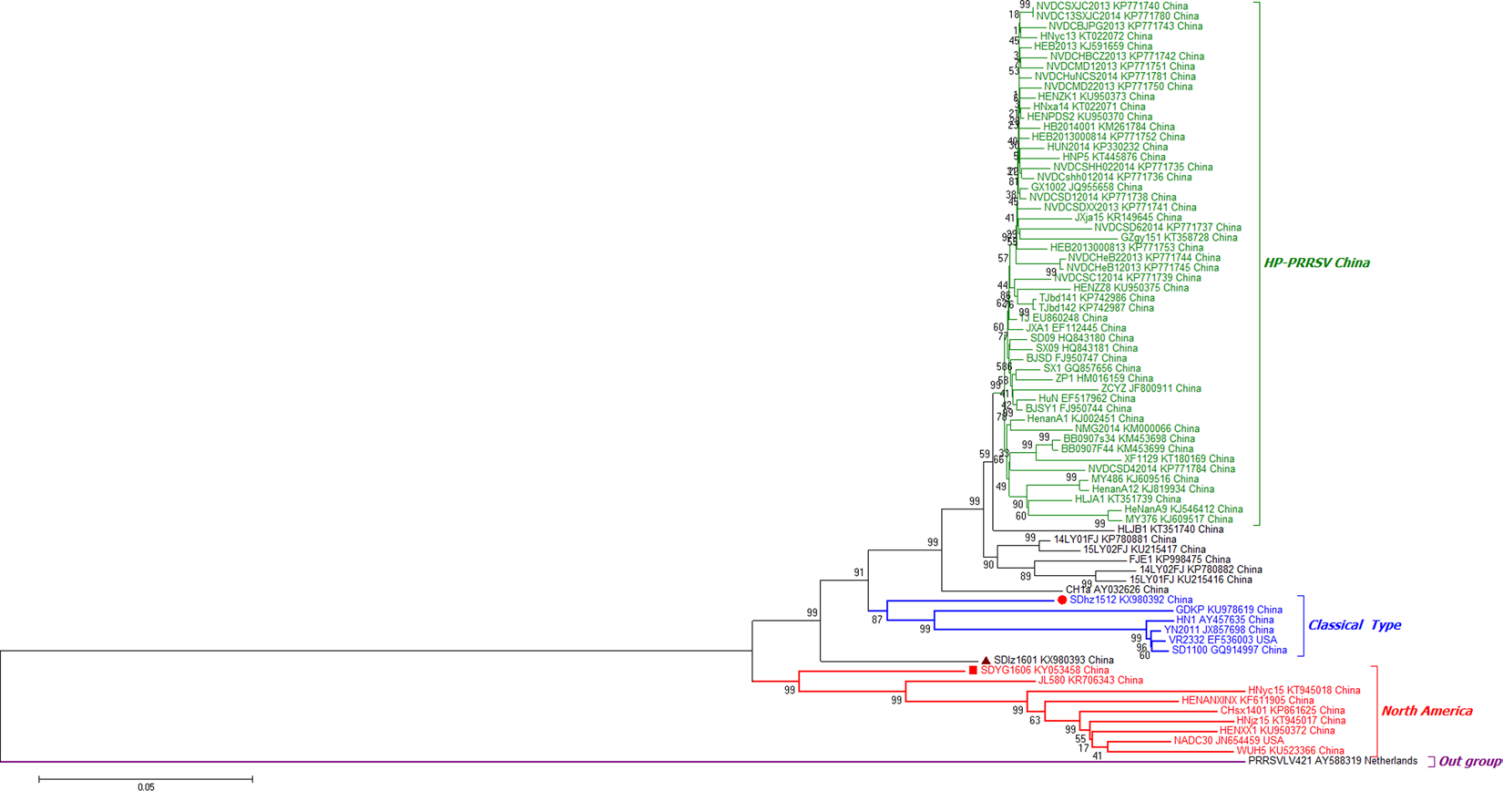
Maximum likelihood tree of whole PRRSV genomes under RaxML (n = 75).

**Figure 7:**
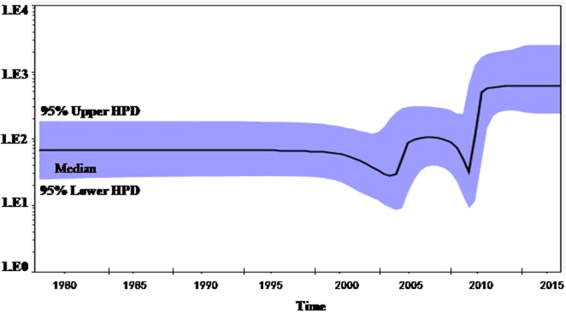
Bayesian skyline plot of global PRRSVs sampled between 2000 and 2016. The dark line in the Bayesian skyline plot shows the estimated effective population size over time. The gray area represents the 95% highest posterior density confidence intervals for this estimate.

To determine the genetic relationship of the HP-PRRSV strains that have recently emerged in China, a phylogenetic tree based on the full genome sequences of SDhz1512, SDlz1601, SDYG1606 and another 73 published PRRSV isolates that were identified between 2013 and 2016 was generated. The phylogenetic analysis of the entire PRRSV genome was performed using RaxML and the BI tree, and its confidence was evaluated with 1000 bootstraps. As shown in Fig. 6, the results showed that genotype II isolates in China could be divided into three sub-genotypes: those closely related to NADC30-like isolates, China classical isolates, and HP-PRRSV isolates. The phylogenetic tree further revealed that SDhz1512, SDlz1601, and SDYG1606 were recombinant strains and had lower identity to other isolates. The complete genome of the three isolates shared only 89–92% identify, which differed from the published NADC30-like strains. This suggests that the virus gained genetic diversity by recombining with local HP-PRRSV, classical strains and North America isolates (represented by NADC30) in China, and these recombinants are becoming more common. The MCC tree revealed, with high resolution, the evolutionary history of NADC30-like viruses and related viruses in China, from which we could infer that NADC30-like PRRSVS were more closely related to NADC30 and were clustered into a separate branch, thus distinguishing them from the HP-PRRSV cluster represented by JXA1 and HuN4. The Bayesian skyline plot revealed two periods in which there was a marked increase in relative genetic diversity (Fig. 7) and whose timing and scale matched well with the epidemiological record of the HP-PRRSV and NADC30 PRRSVS outbreaks. The first growth phase corresponded to the initial HP-PRRSV outbreak in 2005–2010 whereas the second was mainly associated with the new HP-PRRSV variant NADC30-like outbreak in 2013–2017. Recently, outbreaks of this new HP-PRRSV variant have spread all over China.

### PRRSV recombination strains exhibited pathogenicity for piglets

The pigs challenged by PRRSV SDhz1512, SDlz1601 and SDYG1606 strains developed similar clinical signs, such as fever (≥40 °C) and anorexia, and the clinical signs became more severe over the next few days. Three pigs in the SDlz1601 or SDYG1607 strain challenge group had respiratory distress, characterized by dyspnea, tachypnea and coughing, whereas two pigs in the SDhz1512 strain challenge group displayed similar symptoms. The piglets in the control group remained clinically normal throughout the study. The viral titers of the three isolates in sera reached peak levels at 14 days post-inoculation (dpi) (Supplementary Fig. 3), whereas all the pigs in the mock-inoculated control group were negative for PRRSV throughout the entire experimental period. The viruses were recovered from the sera of pigs in the three infected groups, and ORF7 genes were sequenced to confirm they were the original virus.

**Figure 8:**
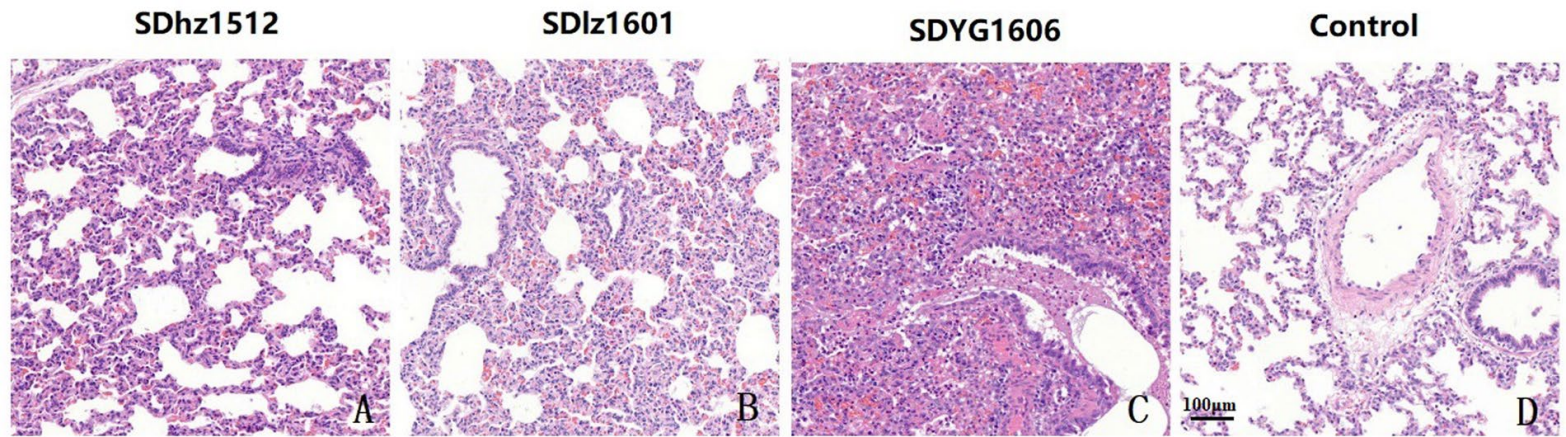
Microscopic lesions examination in lungs of the challenged piglets. (**A**) shows the thickening of the alveolar septa and a small amount of neutrophil infiltration. (**B**) shows thickening of the pulmonary alveolar wall, hyperplasia of alveolar epithelial cells, infiltration of monocytes and macrophages and exfoliated epithelial cells infiltrating in the bronchiole. (**C**) shows that the lung tissue alveolar structure disappeared, infiltration of monocytes, macrophages, neutrophils, and pulmonary parenchymal, and exfoliated epithelial cells infiltrating the bronchiole. (**D**) The lungs of a control pig. Original magnification.

The major gross pathology finding was different levels of interstitial pneumonia in the PRRSV-challenged pigs (Fig. 8). Interstitial pneumonia was characterized by thickening of the alveolar septa and infiltration of infiltrating lymphocytes and macrophages. Necrotic bronchial epithelial cells could also be found in the trachea. No pathological lesion was identified in the control pigs. The morbidity was determined by clinical symptoms and pathological changes (Table 5).

**Table 5:** The morbidity and mortality of pigs used for animal challenge.

Group	Treatment	Number of pigs	Morbidity	Mortality
I	SDhz1512	5	60% (3/5)	0
II	SDlz1601	5	80% (4/5)	20% (1/5)
III	SDYG1606	5	100% (4/5)	20% (1/5)
IV	PAMs culture supernatant	5	0	0

## Discussion

Since 2013, the phenomenon of sow abortion has occurred frequently, resulting in significant losses to the global pig industry. The genetic diversity of HP-PRRSV has greatly increased by rapid evolution and recombination events^14,19,24^. Recently, several outbreaks of NADC30-like PRRSVs were reported in different provinces of China^18^. Strikingly, all NADC30-like strains carry the genetic marker of PRRSV MN184 strains, namely, the exact discontinuous deletions in the NSP2-coding region^20^. Some workers claimed that geographic separation is a factor influencing PRRSV evolution, based on ORF5 and genome sequences^25,26^. Yoon *et al*.^27^ have also reported that there was no immediate relationship between the date or place of collection and the topological distribution of ORF7 in the different PRRSVs. Conversely, Hao X *et al*.^28^ claimed that PRRSV evolution was also based on the ORF7 sequence. However, regarding the phylogenetic relationships among the PRRSVs, our results based on the whole genome sequence data of 73 clinical samples obtained between 2013 and 2016 using RaxML and a BI tree revealed that the Chinese and North American genotypes isolates could be divided into three sub-genotypes using ORF7 primers: those closely related to NADC30 isolates, classical isolates and HP-PRRSV isolates. Moreover, three PRRSV strains (SDlz1601, SDhz1512, and SDYG1606), which had more than 96% homology with NADC30 based on ORF7, were recombinant strains of two or three strains (HP-PRRSV, classical, and NADC30). The PRRSV variants have brought great challenges to the prevention and control of major diseases in the swine industry.

Recombination is not uncommon in PRRSV, and there are more and more reports about the recombination of PRRSVs^5,19^. Recently, Li Y *et al*.^16^ reported PRRSV HNyc15 isolation characterized by NADC30-like recombination with classical PRRSV strain VR2332 and CH-1a between ORF2 and ORF4. Zhao K *et al*.^19^ also indicated that the NADC30-like PRRSV strain from North America has spread to several provinces in China and recombined with local HP-PRRSV strains. Zhao H *et al*.^24^ reported that nine of 28 isolates and one isolate from another laboratory were potential complicated recombinants between the vaccine JXA1-R strains and predominant circulating strains, and the PRRSV recombination rate is increased under the current vaccination pressure. However, our study showed that SDlz1601 and SDYG1606 exhibited a 111 aa deletion at positions 323 to 433 and a 19 aa deletion at positions 506 to 524, which can be considered to be NADC30-like strains. Conversely, the SDhz1512 strain was a recombination strain of NADC30, classical and HP-PRRSV. A 30 aa sequence in the NSP2 gene was detected that was consistent with HP-PRRSV. Accordingly, not only NADC30-like strains but also recombinant strains of NADC30 with classical PRRSV and/or HP-PRRSV have become the current popular trend.

A recent publication indicated that piglets infected with NADC30-like viruses, such as HNjz15, CHsx1401, and FJ1402, showed fever and respiratory disorders, but no death^29,30^. Obviously, the virus NADC30-like pathogenicity is different from the Chinese HP-PRRSV, which is fatal for piglets^31^. In this study, animal challenge was used to determine the relationship between recombination and the virulence of three isolated PRRSV strains. The SDYG1606 strain led to more severe interstitial pneumonia than SDlz1601 although the sequence characteristics of it were similar to NADC30-like strains. The reason for the difference in virulence may result from varied recombination breakpoints. However, the piglet deaths were caused by secondary infection of bacteria. The new variant strain, SDhz1512, which was a triple recombination strain including NADC30, classical and HP-PRRSV, caused more minor clinical symptoms than the NADC30-like strains. Our present data indicated that the virulence of different PRRSV strains with different recombination breakpoints was varied, but the virulence of the recombinant strain with classical PRRSV was slightly weaker.

Bayesian phylodynamic models showed one remarkable improvement compared to traditional methods and could make use of associated epidemiological information to infer genetic relations. These inferences could be used to identify viral dispersion routes that correspond with transportation patterns involving high PRRSV risk^32^. In this study, 75 NSP2 sequences of genotype II PRRSVs from 2000 to 2016 of China obtained from GenBank were used to estimate the evolutionary history of PRRSV in China. The Bayesian skyline plot revealed two periods in which there was a marked increase in relative genetic diversity. The first growth phase corresponded to the initial HP-PRRSV outbreak in 2005~2010, whereas the second growth phase was mainly associated with the NADC30-like outbreak in 2013~2017. It matched well with the epidemiological record of the HP-PRRSV and NADC30 PRRSV outbreaks.

Currently, there are a variety of commercial PRRSV vaccines in the Chinese market. The current modified live vaccines do not provide complete cross-protection against heterologous PRRSV strains^20^. Recent studies suggest that several newly identified virulent PRRSV isolates have been introduced into swine populations through the inoculation of PRRSV-derived inactivated vaccines^33,34^. Recently, Shi M *et al*.^13^ indicated that recombinants appeared to be highly pathogenic, such that the recombination events that generated them either preserved or increased the pathogenicity of the parental strains. Zhao K *et al*.^19^ also confirmed the high pathogenicity of JL580 recombinants between NADC30-like and HP-PRRSV strains in piglet challenge. Several studies reported the outbreaks of NADC30-like PRRSVs in vaccinated pig herds with 30–50% fatality^15^. Thus, the current prevalence of recombinant strains may be the result of PRRSVs escaping vaccine immunization. However, whether the existing vaccine can provide protection for recombinant strains requires further study.

## Material and Methods

### Clinical samples

During the period from June 2014 to April 2017, suspected samples from stillborn piglets, serum samples from diseased sows and piglets, and lungs and lymph nodes of dead or diseased piglets were collected in 13 regions of Shandong Province, China. Clinical tissues were homogenized for RNA extraction and virus isolation, and the remaining samples or serums were kept at −80 °C until use. All animal experimental procedures were approved under the guidelines of the Shandong Province Animal Ethics Committee and conducted in accordance with the accepted policies of our institute and Chinese animal care authorities, in addition to the National Institute of Health Guide for the Care and Use of Laboratory Animals.

### RNA extraction, RT-PCR amplification and sequencing

PRRSV RNAs were extracted using TRZOL reagent (Invitrogen, Carlsbad, CA) and dissolved in nuclease-free water. Reverse transcription PCR (RT-PCR) was performed with a PrimeScript^TM^ One Step RT-PCR Kit (Takara, Dalian, China) according to the manufacturer’s instructions. The primers specific for PRRSV ORF7 encode the nucleocapsid (N) protein. The primer sequences are F: 5′-ATGCCAAATAACAACGG-3′, R: 5′-TGCTGAGGGTGATGCTGT-3′. The PCR cycle parameters were as follows: 50 °C for 30 min, 95 °C for 1 min, 94 °C for 1 min, 52 °C for 1 min and 72 °C for 1 min. A 369 nt amplicon was obtained, analyzed by 1.2% agarose gel electrophoresis, stained with ethidium bromide and visualized under UV light. The PCR products were examined by gel electrophoresis and purified using an Agarose Gel DNA Extraction Kit (BioDev Co., Beijing, China) and then subjected to the Biotechnology Research Center Shandong academy of agricultural sciences (Jinan, China) for Sanger sequencing.

### Isolation and identification of PRRSV

Three strains of PRRSV (SDhz1512, SDlz1601, and SDYG1606) were isolated from serum using porcine alveolar macrophages (PAMs) as previously described^35^. The inoculated cells were maintained at 37 °C in a 5% CO_2_ atmosphere for 72 h. The PRRSV strains were confirmed with an immunofluorescence assay using a monoclonal antibody directed against the PRRSV N protein, which was produced from hybridoma cells derived from Sp2/0 myeloma cells and spleen cells of BALB/c mice immunized with the N protein of PRRSV strain SX-1 (accession no. GQ857656.1).

### Primers designed to determine the complete genome sequence

To determine the complete genomes of SDhz1512, SDlz1601 and SDYG1606 strains, 12 primers were designed on the basis of the genomic sequence of NADC30 available in GenBank (accession No. JN654459). Two Chinese isolates (JXA1 and SD1100) available from the National Center of Biotechnology Information (NCBI) were used as references. The primers used for full genome sequencing of the three isolates are shown in Table 6.

**Table 6:** Primers used for sequencing the full-length genomes of PRRSV isolates.

**Fragment**	**Primer**	**Sequence (5′-3′)**	**Location**
1	PRRSV-F1	atgacgtataggtgttggct	1–20
	PRRSV-R1	atttaccatccggttggcgat	1524–1544
2	PRRSV-F2	acaagtggtatggtgctggga	1327–1347
	PRRSV-R2	gaagcacaacatcccaatcaaagg	2194–2217
3	PRRSV-F3	aaaattgaccagtacctccg	2103–2122
	PRRSV-R3	ggcggtgtctcgagaatcatctt	3483–3505
4	PRRSV-F4	tgatgcgtgaggcatgtgatg	3328–3348
	PRRSV-R4	ggaaagaaaggagctcggaatggaa	4765–4789
5	PRRSV-F5	aaacccatcgcgtatgccc	4602–4620
	PRRSV-R5	gtctttaattatgtggctgcc	6180–6200
6	PRRSV-F6	cattgttacgcgcccttcagg	6071–6091
	PRRSV-R6	gcggccacagcgggtcaagc	7740–7759
7	PRRSV-F7	tcggtatgatgaacgttgacg	7621–7641
	PRRSV-R7	ggtagtttggcatggagggag	9268–9288
8	PRRSV-F8	ttacttcaaaagcggtcaccc	9146–9166
	PRRSV-R8	ggtgaggacttgcccatatt	10582–10601
9	PRRSV-F9	ctgaagaccatctggagatt	10464–10483
	PRRSV-R9	gggcatctcaccatggtatg	11834–11853
10	PRRSV-F10	atttggacccctgcatggccct	11714–11735
	PRRSV-R10	gacaaaaacgccaaccaagc	13051–13070
11	PRRSV-F11	gctcatggtgaattatacggtgt	12864–12886
	PRRSV-R11	cgaaaaattgcatgtcctggcgc	14201–14225
12	PRRSV-F12	tttcctgtgttgactcatattg	14026–14047
	PRRSV-R12	ttttttttttaatttcggccgcatggttct	polyA

### Sequence assembly and alignment

Consensus sequences were assembled using sequence analysis software (DNAStar, version 7.1, Madison WI). Nucleotide and protein identities were searched with BLASTN and BLASTP programs implemented in the BLAST software package (http://www.ncbi.nlm.nih.gov/blast). The percentage of every ORF and most of the derived amino acids were calculated with other isolates using BioEdit software version 7.2.0 (TomHall, Carlsbad, CA, USA). To explore the evolution of the new PRRSV variant in more detail, we downloaded the full genome sequences (n = 73) from GenBank (Table 7). These genomes were aligned in MAFFT versions 7.263 with all other complete sequences from China.

**Table 7:** A total of 76 PRRSV isolates were used in this study.

**No**.	**Virus strain**	**Accession no**.	**Country**	**Province**	**Time**	**No**.	**Virus strain**	**Accession no**.	**Country**	**Province**	**Time**
1	CH1a	AY032626	China	Heilongjiang	2000	39	NVDCHBCZ2013	KP771742	China	Hubei	2013
2	HN1	AY457635	China	Hubei	2003/11/4	40	NVDCBJPG2013	KP771743	China	Beijing	2013
3	JXA1	EF112445	China	Jianxi	2005	41	NVDCHeB22013	KP771744	China	Hebei	2013
4	HuN4	EF517962	China	Hunan	2007	42	NVDCHeB12013	KP771745	China	Hebei	2013
5	VR2332	EF536003	USA	n/a	2005/6/29	43	NVDCMD22013	KP771750	China	n/a	2013
6	TJ	EU860248	China	Tianjin	2006/10/6	44	NVDCMD12013	KP771751	China	n/a	2013
7	BJSY1	FJ950744	China	Beijing	2007/10/7	45	HEB2013000814	KP771752	China	Hebei	2013
8	BJSD	FJ950747	China	Beijing	2007/2/17	46	HEB2013000813	KP771753	China	Hebei	2013
9	SX1	GQ857656	China	Shandong	2008/12/31	47	NVDC13SXJC2014	KP771780	China	Shanxi	2014
10	SD1100	GQ914997	China	Shandong	2008	48	NVDCHuNCS2014	KP771781	China	Hunan	2014
11	ZP1	HM016159	China	Shandong	2009/11/24	49	NVDCSD42014	KP771784	China	Shandong	2014
12	SD09	HQ843180	China	Shandong	2009/4/9	50	14LY01FJ	KP780881	China	Fujian	2014/10/15
13	SX09	HQ843181	China	Shanxi	2009/1/1	51	14LY02FJ	KP780882	China	Fujian	2014/11/12
14	ZCYZ	JF800911	China	Shandong	2009/12/25	52	CHsx1401	KP861625	China	Shanxi	2014/8/30
15	NADC30	JN654459	USA	n/a	2008	53	FJE1	KP998475	China	Fujian	2014
16	GX1002	JQ955658	China	Guangxi	2010	54	JXja15	KR149645	China	Jiangxi	2015
17	YN2011	JX857698	China	Yunnan	2011	55	JL580	KR706343	China	Jilin	2013
18	HENANXINX	KF611905	China	Henan	2013/1/1	56	HNxa14	KT022071	China	Hainan	2014
19	HenanA1	KJ002451	China	Henan	2013/6/30	57	HNyc13	KT022072	China	Hainan	2013
20	HeNanA9	KJ546412	China	Henan	2013/7/4	58	XF1129	KT180169	China	n/a	2013/11/29
21	HEB2013	KJ591659	China	Hebei	2013/9/13	59	HLJA1	KT351739	China	Henan	2013/11/12
22	MY486	KJ609516	China	Henan	2013/7/23	60	HLJB1	KT351740	China	Henan	2013/1/12
23	MY376	KJ609517	China	Henan	2013/7/23	61	GZgy151	KT358728	China	Guangdong	2015
24	HenanA12	KJ819934	China	Henan	2014/4/13	62	HNP5	KT445876	China	Henan	2014/7/10
25	NMG2014	KM000066	China	Neimeng	2014/4/2	63	HNjz15	KT945017	China	Henan	2015
26	HB2014001	KM261784	China	Hubei	2014/3/14	64	HNyc15	KT945018	China	Henan	2015
27	BB0907s34	KM453698	China	Guangxi	2014/2/14	65	15LY01FJ	KU215416	China	Fujian	2015
28	BB0907F44	KM453699	China	Guangxi	2014/2/14	66	15LY02FJ	KU215417	China	Fujian	2015
29	HUN2014	KP330232	China	Hunan	2014/2/20	67	WUH5	KU523366	China	Hubei	2015/9/15
30	TJbd141	KP742986	China	Tianjin	2014	68	HENPDS2	KU950370	China	Henan	2015/4/1
31	TJbd142	KP742987	China	Tianjin	2014	69	HENXX1	KU950372	China	Henan	2014/12/14
32	NVDCSHH022014	KP771735	China	Shanghai	2014	70	HENZK1	KU950373	China	Henan	2014/3/14
33	NVDCshh012014	KP771736	China	Shanghai	2014	71	HENZZ8	KU950375	China	Henan	2015/11/15
34	NVDCSD62014	KP771737	China	Shandong	2014	72	GDKP	KU978619	China	Guangdong	2015/10/12
35	NVDCSD12014	KP771738	China	Shandong	2014	73	SDhz1512	KX980392	China	Shandong	2015/12/1
36	NVDCSC12014	KP771739	China	Sichuan	2014	74	SDlz1601	KX980393	China	Shandong	2016/1/1
37	NVDCSXJC2013	KP771740	China	Shanxi	2013	75	SDYG1606	KY053458	China	Shandong	2016/6/1
38	NVDCSDXX2013	KP771741	China	Shandong	2013	76	PRRSVLV421	AY588319	Netherlands	n/a	38079

n/a: not a vailable.

### NSP2 amino acid sequence alignment, secondary structure and 3D-structure prediction

The NSP2 amino acid sequences of the three PRRSV isolates (SDhz1512, SDlz1601 and SDYG1606) were analyzed by Clustal W software and compared with the standard reference strain sequences. The secondary structure was predicted by the matrix algorithm with the best structure or base pairing using Protein software (DNAStar, version 7.1, Madison WI). The amino acid sequences of the three PRRSV isolates (SDhz1512, SDlz1601 and SDYG1606) and the reference strains (VR2332, JXA1 and NADC30) were submitted to homology modeling of I-TASSER Server^21,22^ for 3D-structure prediction.

### Recombination analysis

RDP4.70 software^36^ was used to test for potential recombination sequences, their probable parental sequences, and the positions of breakpoints. The methods included RDP^37^, GENECONV^38^, MaxChi^39^, Bootscan/Recscan^39^, SiScan^40^ and 3Seq^41^. Default settings were used and the threshold p-value set at 0.05 using the Bonferroni correction. To avoid false positive results, recombination events supported by at least six different methods were considered. The putative recombination events and their breakpoint positions were further validated and confirmed via the SimPlot program (version 3.5.1), which performs similarity comparisons within a 500-bp window sliding along the genome (10 bp step size) using the similarity score and Bootscan plot^42^ based on 26 representative genome sequences. The parental sequences detected were further checked for the presence of recombination. A cluster analysis maximizing the value of x2 was then used to select breakpoints among the clusters^38^. These breakpoints were used to divide the alignment into segments for phylogenetic tree construction.

### Pathogenicity study of the three PRRSV isolated strains

To determine the pathogenicity differences among SDhz1512, SDlz1601 and SDYG1606, an animal experiment was designed. Twenty four-week-old piglets were randomly divided into four groups and maintained in individual rooms. All the piglets were negative for antibodies of porcine circovirus type 2 (PCV2) and PRRSV before the experiment. Each piglet in the PRRSV strain group (SDhz1512, SDlz1601 or SDYG1606) was intranasally administered 2 ml of the virus containing 2 × 10^5^ TCID_50_. Each animal in the control group was given the same dosage of PAM culture supernatant. The viremia of PRRSV-inoculated animals was detected at 0, 3, 7, 14, and 21 dpi by an IFA-microtitration infectivity assay. The animals were euthanized and lung samples were collected for histopathology at 14 dpi.

### Phylogenetic classification of PRRSV

The ORF7 nucleotide phylogenetic and molecular evolutionary analyses of 243 positive PRRSVs were conducted using MEGA version 7, along with 54 sequences of representative PRRSVs available in GenBank from various countries and areas.

Seventy-five complete genomes representing the main PRRSV strains were obtained from GenBank. Multiple sequence alignments were performed in MAFFT version 7.263, and the nucleotide substitution model was evaluated by j-ModelTest v2.1.10 using the Akaike Information Criterion (AIC)^43^. A general-time reversible (GTR) model of nucleotide substitution was used with a gamma-distributed (Γ) rate variation among sites, with a proportion of invariant sites (I). A maximum likelihood (ML) tree was reconstructed using RaxML v8.2.10^44^, and its confidence was evaluated by 1000 bootstraps.

### Time dynamic evolution analysis of PRRSV

According to the description of Shi M *et al*.^13^ and Sook Hee Yoon^26^, 75 NSP2 gene sequences of genotype II PRRSVs from 2000 to 2016 of China obtained from GenBank were used to estimate the evolutionary history of PRRSV in China. Alignments with recombinant regions removed were utilized for phylogenetic analyses using the Bayesian Markov chain Monte Carlo (MCMC) approach available in BEAST v1.8.2^45^. A Bayesian skyline non-parametric coalescent model and uncorrelated lognormal (UCLN) relaxed molecular clock were selected. Bayesian MCMC chains were run for 500 million generations, 10% of which were removed as burn-in, and sampled every 50,000 steps. Convergence and uncertainty in parameter estimates were evaluated by calculating the active sample size^46^ in Tracer v1.6.

### Comments

By submitting a comment you agree to abide by our Terms and Community Guidelines. If you find something abusive or that does not comply with our terms or guidelines, please flag it as inappropriate.

## Electronic supplementary material

Supplementary Information
