# Publisher Correction: Emergence of Different Recombinant Porcine Reproductive and Respiratory Syndrome Viruses, China

**DOI:** 10.1038/s41598-018-25625-z

**Published:** 2018-05-23

**Authors:** Yanyan Liu, Jianda Li, Jie Yang, Hao Zeng, Lihui Guo, Sufang Ren, Wenbo Sun, Zhi Chen, Xiaoyan Cong, Jianli Shi, Lei Chen, Yijun Du, Jun Li, Jinbao Wang, Jiaqiang Wu, Jiang Yu

**Affiliations:** 1 Shandong Key Laboratory of Animal Disease Control and Breeding, Institute of Animal Science and Veterinary Medicine, Shandong Academy of Agricultural Sciences, Jinan, 250100 China; 2 School of Life Sciences, Shandong University, Jinan, 250100 China; 3 School of Life Sciences, Shandong Normal University, Jinan, Jinan, 250014 China; 4 Biotechnology Research Center, Shandong Academy of Agricultural Sciences, Jinan, 250100 China

Correction to: *Scientific Reports* 10.1038/s41598-018-22494-4, published online 7 March 2018

The original version of this Article contained a typographical error in the spelling of the author Yijun Du, which was incorrectly given as Du Yijun. This has now been corrected in the PDF and HTML versions of the Article.

