# Supplementary material for: Emergence of Different Recombinant Porcine Reproductive and Respiratory Syndrome Viruses, China
Source: Sci Rep. 2018 Mar 7;8:4118. doi: 10.1038/s41598-018-22494-4 (PMC5841431; doi:10.1038/s41598-018-22494-4)
Supplement: Supplementary file 1 — Supplementary Information [file 41598_2018_22494_MOESM1_ESM.docx]

**Supplementary Information**

**Emergence of Different Recombinant Porcine Reproductive and Respiratory Syndrome Viruses, China**

Yanyan Liu^a,b,d,1^,Jianda Li^a,1^,Jie Yang^a^, Hao Zeng^a^, Lihui Guo^a^, Sufang Ren^a^, Wenbo Sun^a^, Zhi Chen^a^, Xiaoyan Cong^a^, Jianli Shi^a^, Lei Chen^a^, Yijun Du^a,c^, Jun Li^a,c^, Jinbao Wang^a,b,c^*, Jiaqiang Wu^a,b,c^*, Jiang Yu^a^*

**supplementary Fig.1** Percentage of PRRSV positive samples from 2014 to 2017.


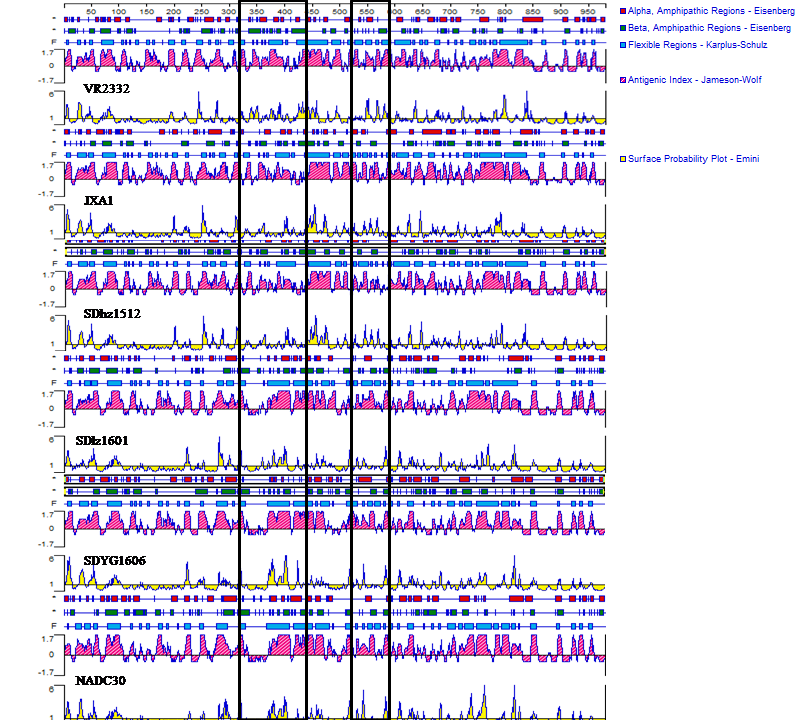


**supplementary Fig.2** Structure prediction of NSP2 protein of PRRSV

SDhz1512, SDlz1601, SDYG1606 and three reference strains with protein software (DNAStar, version 7.1, Madison WI). The black squares indicate the missing amino acid sequence.

**supplementary Fig.3** PRRSV titers in sera of piglets

at different days post-inoculation
